# Earthquake Nowcasting: Retrospective Testing in Greece

**DOI:** 10.3390/e25020379

**Published:** 2023-02-19

**Authors:** Gerasimos Chouliaras, Efthimios S. Skordas, Nicholas V. Sarlis

**Affiliations:** 1 Institute of Geodynamics, National Observatory of Athens, 118 10 Athens, Greece; 2 Section of Condensed Matter Physics and Solid Earth Physics Institute, Department of Physics, National and Kapodistrian University of Athens, Panepistimiopolis Zografos, 157 84 Athens, Greece

**Keywords:** earthquake nowcasting, natural time, earthquake potential score, seismic risk, statistical methods

## Abstract

Earthquake nowcasting (EN) is a modern method of estimating seismic risk by evaluating the progress of the earthquake (EQ) cycle in fault systems. EN evaluation is based on a new concept of time, termed ’natural time’. EN employs natural time, and uniquely estimates seismic risk by means of the earthquake potential score (EPS), which has been found to have useful applications both regionally and globally. Amongst these applications, here we focused on Greece since 2019, for the estimation of the EPS for the largest-magnitude events, $M_{W}$(USGS) ≥ 6, that occurred during our study period: for example, the $M_{W}=$ 6.0 WNW-of-Kissamos EQ on 27 November 2019, the $M_{W}=$ 6.5 off-shore Southern Crete EQ on 2 May 2020, the $M_{W}=$ 7.0 Samos EQ on 30 October 2020, the $M_{W}=$ 6.3 Tyrnavos EQ on 3 March 2021, the $M_{W}=$ 6.0 Arkalohorion Crete EQ on 27 September 2021, and the $M_{W}=$ 6.4 Sitia Crete EQ on 12 October 2021. The results are promising, and reveal that the EPS provides useful information on impending seismicity.

## 1. Introduction

Greece has the highest seismicity in Europe, and statistically produces a large-magnitude (*M*) earthquake (EQ) of the order of *M*6.0 almost every year [1,2,3]: in this sense, Greece is a natural laboratory for seismology, and the short repeat time in the region provides the opportunity to explore changes in regional seismicity rates over “earthquake cycles”. Here, we report the results obtained by the new method of earthquake nowcasting (EN), introduced by Rundle, Turcotte, and coworkers [4]. In this method, to answer the question of the current hazard level of the region, we simply counted the number of small EQs since the last large EQ. Event-counting as a measure of “time”, rather than clock time, is known as “natural time”, a concept that was introduced by Varotsos et al. [5,6,7,8,9]. Rundle et al. [4] pointed out that the use of natural time has the following two advantages when applied to EQ seismicity: firstly, it is not necessary to decluster the aftershocks, as natural time is uniformly valid when aftershocks dominate, when background seismicity dominates, and when both contribute; secondly, in computing nowcasts, the concept of natural time—counts of small EQs—is used as a measure of the accumulation of stress and strain between large EQs in a defined geographic region.

In other words, the basis of nowcast is the use of natural time. There exist two advantages, as mentioned, to using natural time: the first is that it is not necessary to separate the aftershocks from the backgroung seismicity; the second is that only the natural interevent count statistics are used, rather than the seismicity rate, which involves conventional (clock) time as well. The nowcasting method considers an “EQ cycle” as the recurring large EQs in a large seismically active region comprising several active faults, rather than the traditional focus on recurring events on individual faults. Following Pasari [10] (see also Pasari et al. [11]), we may state that, though the idea of natural time is unique in its characteristics, the notion of “EQ cycle” has been applied in many earlier seismological studies [12,13,14].

**Figure 1:**
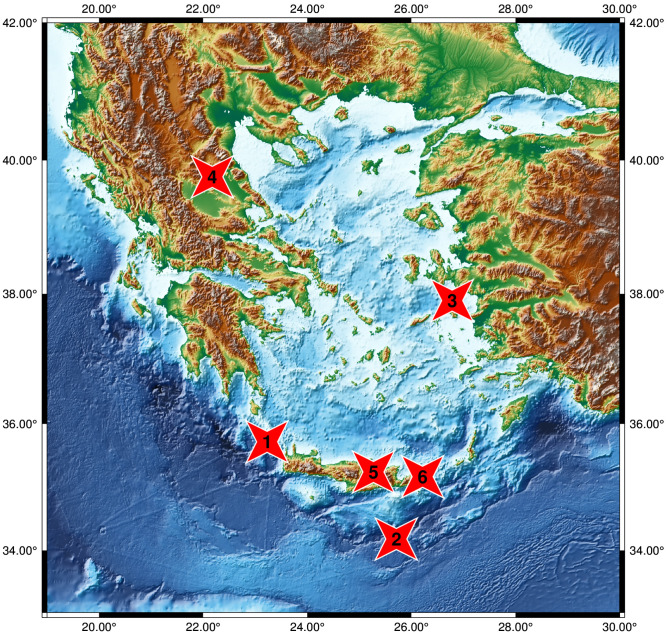
Map with the epicenters (red stars) of the six events with $M_{W}$(USGS) ≥ 6 during the period 1 January 2019 to 6 February 2023, in Greece. The stars correspond, from top to bottom, to the $M_{W}=$ 6.3 Tyrnavos EQ on 3 March 2021, the $M_{W}=$ 7.0 Samos EQ on 30 October 2020, the $M_{W}=$ 6.0 WNW-of-Kissamos EQ on 27 November 2019, the $M_{W}=$ 6.0 Arkalohorion Crete EQ on 27 September 2021, the $M_{W}=$ 6.4 Sitia Crete EQ on 12 October 2021, and the $M_{W}=$ 6.5 off-shore Southern Crete EQ on 2 May 2020, respectively, with their corresponding numbers in Table 1. Reference [26] was used to produce this map.

The method of EN has been widely and successfully applied in the estimation of the seismic risk in global megacities [15], in the study of induced seismicity [16], in the study of temporal clustering of global EQs [17], in clarifying the role of small EQ bursts in the dynamics associated with large EQs [18], in understanding the complex dynamics of EQ faults [19], in identifying the current state of the “EQ cycle” [20,21,22], in nowcasting avalanches [23], in the Olami–Feder–Christensen model [24], and, very recently, in the study of volcanic eruptions [25]. Here, we studied the largest-magnitude events, with $M_{W}$(USGS) ≥ 6, that occurred in Greece between 1 January 2019 and 6 February 2023 (see Figure 1 and Table 1), by means of the earthquake potential score (EPS) (see below).

In general, natural time analysis (NTA), which was reviewed in [9] and, more recently, in [27], reveals the dynamical evolution of a complex system, and identifies when it enters a critical stage. As such, NTA is able to play a key role in predicting impending catastrophic events, such as the occurrence of large EQs: in this sense, it was applied in cases of EQs in Greece [5,7,8,28,29,30,31], Japan [32,33,34], the USA [35,36], Mexico [37,38,39], the Eastern Mediterranean [40,41], and globally [42,43,44]. We note that NTA allows the introduction [5,45,46] of an entropy, *S*, which is a dynamic entropy [47] that exhibits positivity, concavity, and Lesche [48,49] experimental stability. Complexity measures [50] based on natural time entropy, and on *S* itself, have recently been applied to the study of EQs in Japan [51,52,53,54] and Mexico [55,56], with promising results [57]. Specifically, as mentioned very recently in the Preface of [27], two quantities, which are described below, have emerged from natural time analysis, to play an important role in estimating *if* and *when* the critical point (mainshock, the new phase) is approached.

First, the order parameter $\kappa_{1}$ of seismicity: its value (=0.070) identifies when the system enters the critical stage; as for its fluctuations, their minimum marks the time when the Seismic Electric Signals (SES) [58,59,60,61,62,63] activities initiate [64]. The SES amplitude is important, as it enables the estimation of the magnitude of the impending mainshock, and the epicentral area is estimated on the basis of the SES selectivity map of the station, which records the relevant SES (on this basis, a successful prediction was issued [29,31,65] for an $M_{W}=6.4$ EQ that occurred on 8 June 2008 in the Andravida area in Greece).

Secondly, the entropy change, ΔS, under time reversal: its value–when minimized a few months in advance–marks the initiation of precursory phenomena; as for its fluctuations (when the minimum of ΔS appears), they exhibit an evident increase, pointing to the time when the EQ preparation starts, as explained by the physical model that inspired the SES research [63].

These two quantities were used for the study of precursory phenomena before the following two major EQs: the $M_{W}=$ 9.0 Tohoku EQ on 11 March 2011, which was the largest event ever recorded in Japan; and the $M_{W}=8.2$ Chiapas EQ that occurred on 7 September 2017, which was the largest EQ in Mexico for more than a century.

The present paper is organized as follows: in Section 2, the data used and the methods employed are described; in Section 3, the results are presented, in brief; a discussion follows in Section 4; finally, Section 5 summarizes the conclusions.

## 2. Data and Methods

For the present analysis, the events reported since 1 January 1980 in the United States Geological Survey (USGS) earthquake catalog—as described in [66] (see also https://earthquake.usgs.gov/fdsnws/event/1/)—were used (accessed on 6 February 2023). Additionally, the EQs (since 24 February 1964) of the National Observatory of Athens (NOA) earthquake catalog [2], available at https://www.gein.noa.gr/en/services-products/earthquake-catalogs/, were considered (accessed on 6 February 2023).

As concerns notation, the generic symbol *M* stands for the EQ magnitude, i.e., the preferred magnitude either in the USGS or the NOA catalog, while $M_{W}$(USGS), or simply $M_{W}$, is used for the moment magnitude [67] of the USGS. The local EQ magnitude reported by the NOA is denoted by ML(NOA), when needed.

**Figure 2:**
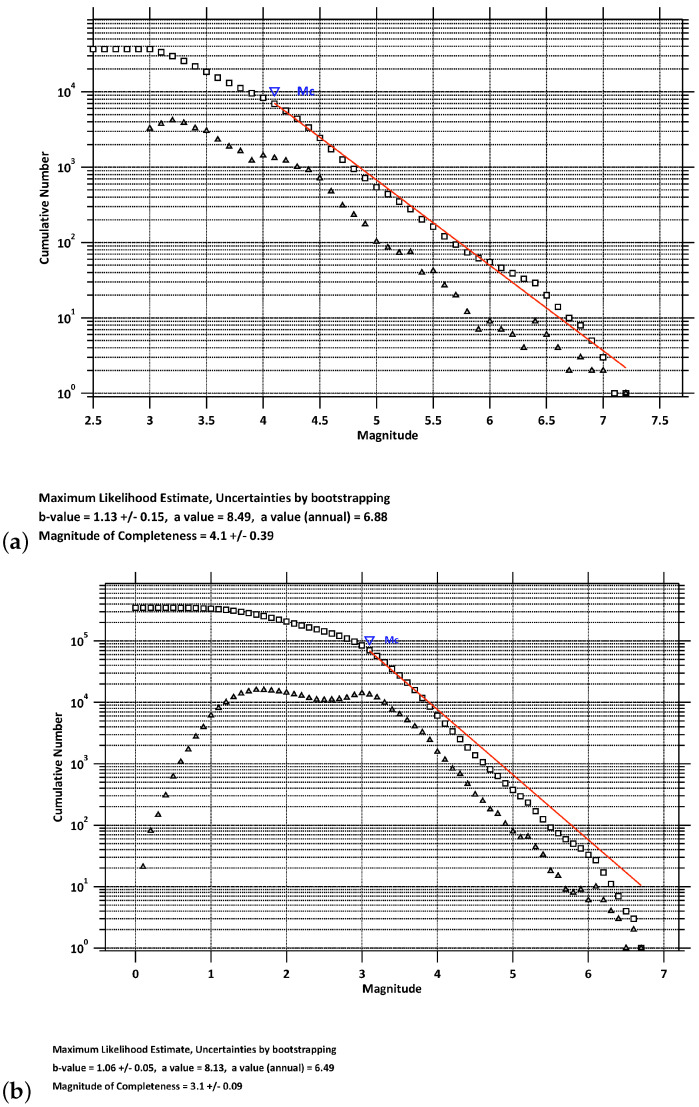
Determination of $M_{c}$ for: (**a**) the USGS catalog; (**b**) the NOA catalog. The squares depict N(>M), while the triangles depict the number of EQs that occurred during the concerned period, within a ±0.05 range of magnitude.

We now examine, within the regions $N_{18}^{56}E_{1}^{45}$ and $N_{34}^{42}E_{19}^{30}$ of the USGS and the NOA catalogs, respectively, the completeness magnitude $M_{c}$ of the Gutenberg–Richter (GR) law [68] of EQs. The latter states that:

(1) $$\log_{10}\left[N\left(>M\right)\right]=a-bM,$$

where N(>M) is the number of EQs exceeding a magnitude *M*, while *a* is related to the total seismicity rate of the region, and *b*, which should usually be observed to be near 1, is set by the level of seismicity in the region [69]. A catalog is assumed to be complete (in a certain space–time region), at least for a magnitude $M_{c}$, when every earthquake of magnitude at least $M_{c}$ in that space–time region is present in the catalog, assurance of which is secured by the validity of Equation (1) (see, e.g., [2,70]). The maximum likelihood estimation [71] for the GR law is shown in Figure 2 for the aforementioned regions: this results in b=1.13±0.15 with $M_{c}=4.1$, and b=1.06±0.05 with $M_{c}=3.1$ for the USGS and NOA catalogs, respectively. In simple words, a GR law *b*-value equal to 1 means that, when assuming ≈ 1000 M≥ 3 events to have occurred in a spatial region since the last M≥ 6 EQ, another M≥ 6 EQ should be expected soon.

Earthquake nowcasting [4] uses an EQ catalog to calculate, from the number *n* of “small” EQs —defined as those with magnitude $M<M_{\lambda }$, but above a threshold $M_{\sigma }$, i.e., $M\in [M_{\sigma },M_{\lambda })$— that occurred after the last “large” $M\geq M_{\lambda }$ EQ, the level of hazard for such a “large” EQ: thus, the number *n* stands for the waiting natural time or interoccurrence natural time [39,41,44]. The current value, n(t), in a given region since the occurrence of the last “large” EQ is compared with the cumulative distribution function (CDF) P[n<n(t)] obtained, when ensuring that we have enough data to span at least 20 or more “large” EQ cycles. The EPS equals the current CDF value, i.e., EPS = P[n<n(t)], and is therefore a unique measure of the current level of hazard, which assigns a number between 0 and 100%. For the calculation of the CDF, the EQs with depths smaller than a certain value *D* within the region are considered. The seismic risk for various cities throughout the world has been estimated [4,10,15,72,73,74,75] by first calculating the CDF P[n<n(t)] within a large region—such as $N_{18}^{56}E_{1}^{45}$ and $N_{34}^{42}E_{19}^{30}$ in our case—and comparing it with the number $\tilde{n}$ of the “small” EQs around the city: these being the EQs that had occurred within a circular area of epicentral distances, r<R, since the occurrence of the last “large” EQ there. As EQs exhibit ergodicity [76,77,78], Rundle et al. [15,73] proposed that the seismic risk for the city can be found by substituting $\tilde{n}$ for n(t) in the aforementioned CDF, i.e.,

(2) $$\mathrm{EPS}=P[n<\tilde{n}].$$

**Figure 3:**
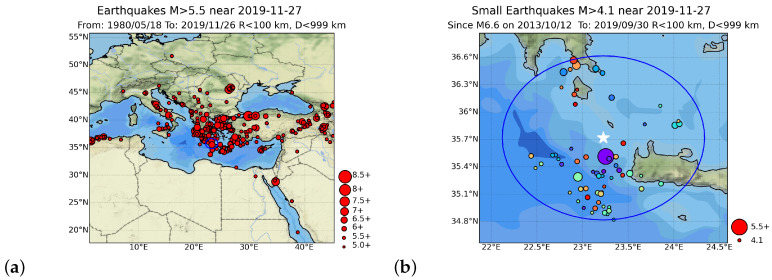
Analysis example for the WNW-of-Kissamos EQ on 27 November 2019: $M_{W}$ = 6.0; ML(NOA) = 6.1 within 100 km radius of the epicenter. Panel (**a**) depicts the regional seismicity during the period from 18 May 1980 to 26 November 2019, just before the EQ occurrence. Panel (**b**) depicts the local seismicity since the last $M_{W}$ = 6.6 EQ, on 12 December 2013.

In other words, whereas the statistics come from the large map region, (see, e.g., Figure 3a), the current count $\tilde{n}$ of “small” EQs comes from “small” events that occur within the circular region of radius, (see, e.g., Figure 3b). We note that EN calculations are not based on a model, but on the empirical CDF obtained. Exact details of the rankings in EN may be sensitive to the radius *R* and depth *D* of the circular region, the spatial extent of the large region in the map, and the quality and completeness of the catalog data. It is possible to have an EPS = 100%, meaning that a large EQ is overdue, based on historical catalog statistics. Weibull statistics has been used in many cases [10,39,41,44,74], to approximate the empirical CDF, in order to convert $\tilde{n}$ into a probability.

Here, we employed the most recent version of the Megacities Earthquake Nowcasting software [66] (see also [15,73]), which used the empirical CDF estimated in the large region, as shown in Figure 3a, to calculate the EPS on the basis of Equation (2), and by assuming $M_{\sigma }=M_{c}$ in each catalog case. We also considered, as the center of the circular region, the epicenter of each of the strong EQs compiled in Table 1. Rundle et al. [15,73], when considering in their Figure 2 the large area $N_{12}^{35}E_{29}^{47}$, in order to estimate the EPS for EQs of magnitude greater than or equal to $M_{\lambda }$ = 6.5, assumed $R_{0}\;=$ 400 km and $D_{0}=200$ km around the capital of Greece, i.e., Athens. Following [72], and taking into account that the $10^{0.5M_{m}}$ scaling is expected to hold for the linear size of both the aftershock [79] and the foreshock [80] zone of a mainshock of magnitude $M_{m}$, we assumed that

(3) $$R\left(M_{\lambda }\right)=R_{0}\times 10^{0.5(M_{\lambda }-6.5)}.$$

This led to R≈ 126 km for the case of $M_{\lambda }=5.5$, which was further truncated to R= 100 km for simplicity, and was used throughout this study. Moreover, the value of *D* was not considered essential, as EQs deeper than $D_{0}$ are extremely rare in Greece: thus, we set D=999 km.

Finally, we briefly describe the self-consistent method of producing average EPS maps, also termed 〈EPS〉 maps, using a coarse-grain radius, *R*: this was introduced and applied to the Eastern Mediterranean area $N_{25}^{50}E_{5}^{46}$ in [41], and was further developed by Perez-Oregon et al. [39] and Christopoulos et al. [44]. To construct such a map, one first estimates the EPS for disks of radius *R* centered at the points $\left(x_{ij},y_{ij}\right)$ of a lattice (here, of a unit cell $0.25^{{}^{\circ}}\times 0.25^{{}^{\circ}}$), to obtain the EPS${}_{ij}$; one then averages, for each point $\left(x_{i_{0}j_{0}},y_{i_{0}j_{0}}\right)$, the estimated EPS values within the same radius, *R*, i.e.,

(4) $$\langle \mathrm{EPS}\rangle \left(x_{i_{0}j_{0}},y_{i_{0}j_{0}}\right)\equiv \frac{1}{N}\sum_{i,j}^{d(x_{i_{0}j_{0}},y_{i_{0}j_{0}};x_{ij},y_{ij})\leq R}{EPS}_{ij},$$

where the summation is restricted to the lattice points whose distances $d(x_{i_{0}j_{0}},y_{i_{0}j_{0}};x_{ij},y_{ij})$ from the observation point are smaller than or equal to *R*, with *N* standing for the number of lattice points included in the sum.

## 3. Results

**Figure 4:**
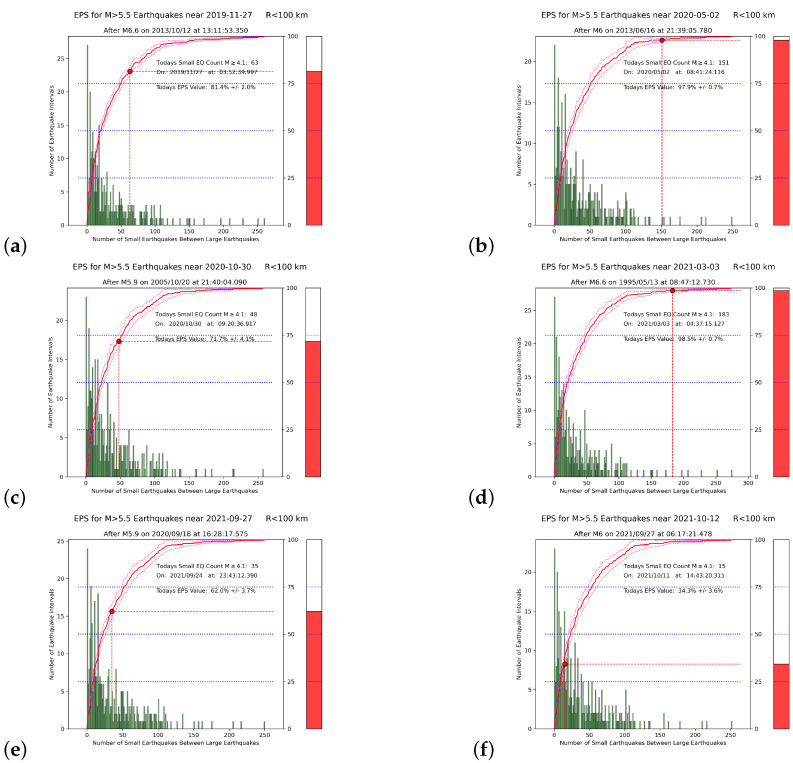
Earthquake nowcasts, when using the USGS catalog with $M_{\sigma }=4.1$, within the 100 km circle around each of the Figure 1 EQs in Greece. Panels (**a**–**f**) correspond to the Kissamos, off-shore Southern Crete, Samos, Tyrnavos, Arkalohorion, and Sitia Crete EQs, respectively, in Table 1. Green bars comprise the histogram of the number of small EQs within each circular region. The red curve is the experimental CDF, and the red dot corresponds to its value at the current number, $\tilde{n}$, of “small” EQs since the last large event. The thermometer on the right side is a pictorial representation of the current state of the “EQ cycle”.

Using the USGS catalog, we studied all six EQs with $M_{W}\geq$ 6 during the period from 1 January 2019 to 6 February 2023. The results for $M_{\sigma }\;=\;M_{c}=4.1$, $M_{\lambda }\;=\;5.5$, and R=100 km are shown in Figure 4. The corresponding EPS just before the EQ occurrences was inserted into the last column of Table 1.

**Figure 5:**
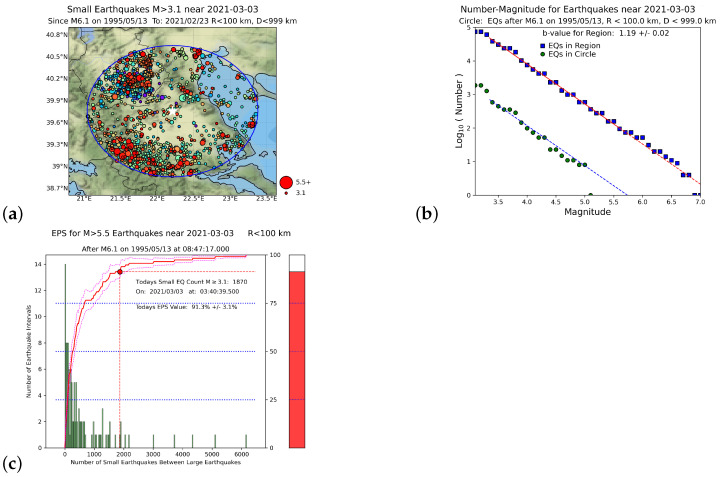
EN results for the Tyrnavos EQ on 3 March 2021, using the NOA catalog with $M_{\sigma }$ = 3.1: (**a**) map of EQs near Tyrnavos, Greece having magnitude M≥3.1 since 1995; the blue circle centered at the Tyrnavos EQ epicenter (white star) has radius R= 100 km; (**b**) GR law statistics; the upper blue square symbols are all EQs in the region $N_{34}^{42}E_{19}^{30}$ as a whole; the lower green circles are all EQs inside the circular area shown in (**a**) since the last ML(NOA) = 6.1 EQ on 13 May 1995, i.e., the date of the $M_{W}$ = 6.6 EQ at Grevena–Kozani; (**c**) Nowcast for $M_{\lambda }=5.5$; the green bars are a histogram of the number of small EQs within the circular region; the red curve is the experimental CDF, and the red dot corresponds to its value at the current number $\tilde{n}$ of “small” EQs since the last “large” ML(NOA) = 6.1 event on 13 May 1995, inside the blue circle of panel (**a**); the thermometer on the right side is a pictorial representation of the current state of the “EQ cycle”.

As an example, we also present, in Figure 5, the EN results obtained when using the NOA catalog for the Tyrnavos EQ. Since the unification in 2012 of the Greek national seismological network, the NOA catalog has been substantially improved, in terms of both the detectability of seismic events and the magnitude of completeness. Recent EQ activity has been well-monitored in Greece, and the EQ catalog of the NOA is the richest database for investigating the associated seismicity patterns [1,2,81]. As mentioned, for the NOA catalog, $M_{c}$ = 3.1, (see Figure 2b): hence, we considered, in the NOA case, $M_{\sigma }=M_{c}=3.1$, and the corresponding EPS became 91.3% (see Figure 5c), in comparison to 98.5% when using the USGS catalog (see Figure 4d).

## 4. Discussion

In physics, event count models are called natural time models, as counts of small events represent a physical or natural time scale characterizing the system dynamics. The fundamental assumption [82] is that a deficiency of large EQs within a local region contained within a seismically active larger region will eventually be filled in by the occurrence of large EQs. The idea is that the statistics of smaller regions over long times will be the same as the statistics of the larger region over large spatial domains and long times. Small events can therefore be used as a kind of “clock” that marks the “natural time” between the large events.

An inspection of Table 1 reveals that in five out of the six strong EQs studied, the EPS values exceed 50%: this result is compatible with the EPS value obtained in [72], just before the occurrence of the $M_{W}=6.8$ EQ that struck Western Greece on 25 October 2018, which had an epicenter at 37.5${}^{{}^{\circ}}$ N 20.6${}^{{}^{\circ}}$ E, i.e., close to the Zakynthos island [83]. In that study, the SES—which, we recall, are low-frequency f(≤1 Hz) electric signals that precede [30,31,35,59] strong EQs— was recorded on 2 October 2018, in the form of SES activity [60,61], i.e., a series of transient variations of the Earth’s electric field, recorded within a short time interval, as shown in Figure 2 of [72]: this SES activity was combined with NTA [5,7,9,29,84] and EN to show that a strong EQ was imminent after 18 October 2018 in Western Greece, and inside the gray shaded area shown in Figure 1 of [72]. The latter area corresponded to the selectivity map [60,61] of the station that recorded the SES, and was determined [29] on the basis of the successful predictions [30,31] of all the $M_{W}\geq 6.4$ EQs that struck Western Greece in 2008. In that study, Equation (3) was taken into account, and a circular area of radius R=225 km around the city of Patras was studied for $M_{\lambda }=6.0$ (see Figure 1 of [72]): the actual epicenter [83] lay inside both the selectivity map and this circular area. The EPS value obtained [72] using the United States National EQ Information Center (NEIC) Preliminary Determination of Epicenters (PDE) catalog—which is available from USGS at https://earthquake.usgs.gov/earthquakes/search/ (accessed on 6 February 2023)— resulted in 80% (see Figure 4 of [72]). If we further consider that this $M_{W}=$ 6.8 EQ was the single EQ in 2018 of magnitude $M_{W}\geq 6$ in Greece, we find that in six out of the seven cases of EQs with $M_{W}\geq 6$ in Greece since 1 January 2018—i.e., more than four years—the EPS exceeded 50% just before the corresponding EQ occurrences.

**Figure 6:**
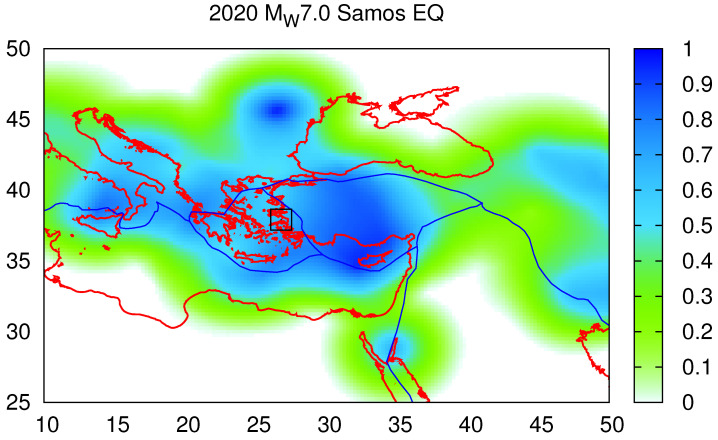
Average EPS map, calculated on the basis of the data available on 30 October 2020, just before the $M_{W}=$ 7.0 Samos EQ, with a coarse-grain radius R=250 km, in a fashion similar to those presented in Figures 4 and 6 of [41]. The EQ epicenter is indicated by the black square, and the value of the EPS at the grid point closest to this epicenter location is 63.2%.

We now turn to a comparison with the recent work of Varotsos et al. [41], in which complex EQ networks based on similar activity patterns [85], together with NTA [40] and EN, were combined, in an attempt to obtain information on the epicenter location of an impending destructive ($M_{W}\geq 7.1$) EQ, in the form of the self-consistent 〈EPS〉 maps (Section 2), using the NEIC PDE catalog, $M_{\lambda }$ = 6.0, $M_{\sigma }$ = 4.0, and R= 250 km$[\approx R\left(M_{\lambda }=6.0\right)=225$ km]. Here, as a comparison, we present in Figure 6 the 〈EPS〉 map corresponding to the $M_{W}=7.0$ Samos EQ of Table 1, which was the strongest EQ in our study. We observed that the value of the EPS closest to the epicenter was 63.2%, which does not differ much from the value of 71.7% reported in Table 1, in the sense that they both exceeded 50% and differed less than 10%. We can also compare the aforementioned value of 80% reported in [72] with the value of 71% reported as the EPS closest to the epicenter location of the corresponding 〈EPS〉 map in [41] (see their Table 2, for the 2018 $M_{W}=$ 6.8 Zakynthos EQ): hence, we can see that there is consistency (within 10%) of the EPS values reported in the earlier EN studies in Greece [41,72], meaning that the EPS can identify the progress of the “EQ cycle” in a consistent way, when employing Equation (3). We recall that, as mentioned in Section 2, this Equation correctly reflects the fault system dynamics [79,80].

For the Tyrnavos EQ, the same consistency was observed when comparing the EPS values 98.5% and 91.3%, obtained (see Figure 4d and Figure 5c) when using either the USGS or the NOA catalog, respectively. This was the first time that a local catalog, i.e., the NOA catalog, was used for EN in Greece. The results show remarkable consistency, implying that in future studies the NOA catalog will be particularly useful for EN in Greece.

**Figure 7:**
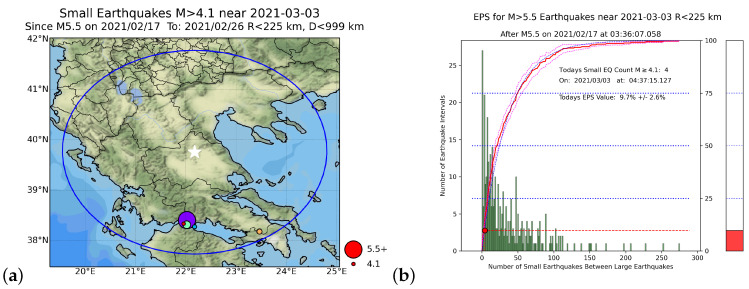
EN results for the Tyrnavos EQ on 3 March 2021, using the USGS catalog ($M_{c}$ = 4.1) but with R=225 km: (**a**) map of EQs having magnitude M≥4.1 near Tyrnavos, Greece since 17 February 2021; the blue circle centered on the Tyrnavos EQ epicenter (white star) has radius R= 225 km; (**b**) Nowcast for $M_{\lambda }=5.5$; the green bars are a histogram of the number of small EQs within the circular region; the red curve is the experimental CDF, and the red dot corresponds to its value at the current number $\tilde{n}$ of “small” EQs since the last “large” $M_{W}=5.5$ event on 17 February 2021 at 38.4${}^{{}^{\circ}}$ N 22.0${}^{{}^{\circ}}$ E inside the blue circle of panel (**a**); the thermometer on the right side is a pictorial representation of the current state of the “EQ cycle”.

In order to further examine the importance of Equation (3), we present in Figure 7 the results obtained when using the USGS catalog for the Tyrnavos EQ, but when employing R=225 km. We observe that the corresponding EPS was 9.7% in comparison to the aforementioned value of 98.5% when R=100 km. Moreover, we established that the EPS was 99.4% when R=126 km, as it results from Equation (3) for $M_{\lambda }=5.5$, thus differing less than 1% from that obtained for R=100 km (cf. the latter value of *R*, as mentioned, is used for the EPS calculations throughout this paper for simplicity). Hence, we conclude that when violating Equation (3), incompatible values of the EPS may appear.

When considering all the above, we find that the results reported here are promising, and reveal that the EPS provides useful information on impending seismicity. Of course, a better understanding of EQ source physics and interaction, as well as longer EQ observation with low magnitudes of completeness, may provide valuable information on precursory seismicity patterns, and help in finding the ultimate goal of EQ forecasting research.

## 5. Conclusions

In the present work, we found that EN, when using either the USGS or the NOA catalog, provides useful information about the occurrence of strong $M_{W}\geq 6$ EQs in Greece. The calculated EPS values exhibited consistency within 10%, when employing different $M_{\lambda }$, which, in our opinion, was due to the application of Equation (3): this should be taken into account in future works.

In summary, we found that in six out of the seven cases of strong EQs ($M_{W}\geq 6$) in Greece since 2018, the EPS exceeded the value of 50% close to the epicenter location: hence, we can say that they were overdue. All these cases concerned EQs with magnitudes in the range $6.0\leq M_{W}\leq 7.0$, and should be considered in comparison to the previous EN studies [41,72] in Greece.

## Figures and Tables

**Table 1:** All EQs in Greece with $M_{W}$(USGS) ≥ 6 from 1 January 2019 to 6 February 2023: for the location of their epicenters, see the map of Figure 1. In the last column, we inserted the EPS, calculated on the basis of the USGS catalog (see Section 3).

Serial Number	EQ Name	EQ Date	Lat, ${}^{{}^{\circ}}$N	Long, ${}^{{}^{\circ}}$E	$M_{W}$(USGS)	ML(NOA)	EPS (%)
1	WNW-of-Kissamos	27 November 2019	35.7	23.2	6.0	6.1	81.4
2	Off-shore Southern Crete	2 May 2020	34.2	25.7	6.5	6.0	97.9
3	Samos	30 October 2020	37.9	26.8	7.0	6.7	71.7
4	Tyrnavos	3 March 2021	39.8	22.2	6.3	5.9	98.5
5	Arkalohorion Crete	27 September 2021	35.2	25.3	6.0	5.8	62.0
6	Sitia Crete	12 October 2021	35.2	26.2	6.4	6.3	34.3

## Data Availability

All seismological data used are publicly available.
